# “I Don’t Know Where I Have to Knock for Support”: A Mixed-Methods Study on Perceptions and Experiences of Single Mothers Raising Children in the Democratic Republic of Congo

**DOI:** 10.3390/ijerph181910399

**Published:** 2021-10-02

**Authors:** Mikyla A. Callaghan, Dédé Watchiba, Eva Purkey, Colleen M. Davison, Heather M. Aldersey, Susan A. Bartels

**Affiliations:** 1 Department of Biomedical and Molecular Sciences, Queen’s University, Kingston, ON K7L 3N6, Canada; 17mac5@queensu.ca; 2 Department of Political Science, University of Kinshasa, Kinshasa, Democratic Republic of the Congo; dedewatchiba@yahoo.com; 3 Department of Family Medicine, Queen’s University, Kingston, ON K7L 3G2, Canada; eva.purkey@queensu.ca; 4 Department of Public Health Sciences, Queen’s University, Kingston, ON K7L 3N6, Canada; davisonc@queensu.ca; 5 Department of Global Development Studies, Queen’s University, Kingston, ON K7L 3N6, Canada; 6 School of Rehabilitation Therapy, Queen’s University, Kingston, ON K7L 3N6, Canada; hma@queensu.ca; 7 Department of Emergency Medicine, Queen’s University, Kingston, ON K7L 4V7, Canada

**Keywords:** Democratic Republic of Congo, parenting challenges, single mother, single mother parenting, social support services, women and children

## Abstract

*Introduction and Objectives*: It is well-documented that single mothers in sub-Saharan Africa face unique psychosocial challenges which can lead to child health and developmental disadvantages, often impacting life trajectories for both the mother and child. Years of instability, conflict, and widespread poor governance within the Democratic Republic of Congo (DRC) have resulted in magnified challenges for parents, making it more difficult to provide supportive and effective parenting. To address gaps in knowledge regarding the specific challenges experienced and adaptations made among single mothers raising children in contexts of adversity, the present study aimed to investigate the phenomenon of single mother parenting in the DRC. *Methods*: Cognitive Edge SenseMaker, a mixed-method data collection tool, was used to collect self-interpreted narratives among parents in eastern DRC. Quantitative SenseMaker data were uploaded into Tableau, a data organization and analysis tool, to visualize differences in response patterns between single mother (*n* = 263) and two-parent family study participants (*n* = 182). Single mother micronarratives (*n* = 251) were then coded line-by-line and analyzed thematically. Qualitative themes identified in the single mother micronarratives were used to facilitate a deeper and more nuanced understanding of key quantitative SenseMaker findings. *Findings and Conclusions*: Our study found that single mothers experienced immense challenges raising children in the DRC, including financial-, health- and parenting-related hardships. Single mothers described negative emotions and higher levels of household adversity while providing for their children in situations of extreme poverty compared to two-parent family respondents. Self-reliance was exhibited among most single mothers in an attempt to overcome challenges, primarily financial barriers, and to prioritize the health and well-being of their children. However, many children still lacked access to sufficient food, education, and healthcare. Limited governmental and social security support for single mothers was identified as contributing to heightened challenges and the self-reliance observed among single mothers. Findings emphasize that additional research and attention should be directed towards identifying the specific needs of, and available resources for, single mothers in different localities in an effort to inform policies and programs that best support families.

## 1. Introduction

The Democratic Republic of Congo (DRC) has experienced recurrent conflict and violence, as well as political and economic instability for decades. Women have been disproportionately affected by DRC’s instability, with oppressive and discriminatory gender norms having decreased opportunities for women with respect to education, employment, participation in politics, and access to resources [1]. It is estimated that 61.2% of women in the DRC live under the poverty threshold versus 51.3% of men [2]. Moreover, with widespread and pervasive gender-based violence in the DRC, approximately 68.2% of women report experiencing lifetime physical, sexual, or emotional violence [2].

In several sub-Saharan African contexts, high rates of abuse and neglect by men, low marriage rates, and little paternal involvement of fathers in their children’s lives have been described [3,4,5]. Within these contexts, children most often spend the critical early years of their lives with a single mother. In the DRC, divorce and separation for women aged 15–49 increased by over 50% between 1984 and 2007 [6]. However, other factors leading to single motherhood, such as extramarital childbirth, separation, widowhood, and sexual violence have not been well quantified; thus, the prevalence and determinants of single motherhood in the DRC are still largely unknown.

### 1.1. Single Motherhood in Sub-Saharan Africa

Research suggests that two-parent households provide the greatest degree of care for children in sub-Saharan Africa [5,7,8], which may be due to distinct gendered-household roles of mothers and fathers in African family contexts [9]. Disruptions that result in one parent not fulfilling caregiving responsibilities can greatly affect the other caregiver’s ability to provide for their children, potentially hindering children’s access to healthcare, nutrition, and/or schooling [7,10].

Studies have indicated high levels of premarital childbirth in sub-Saharan Africa, accounting for as many as one-half of births among women aged 12–26 in some regions, and one in four births among women aged 15–19 in others [11,12]. Out-of-wedlock motherhood may present unique economic, logistical, and societal challenges. For instance, Smith-Greenway (2016) found that under-educated mothers who gave birth premaritally may have fewer resources themselves and have lower-resourced families to turn to for support [13]. Single motherhood due to premarital childbirth may also result in negative community perceptions and stigma. A recent study conducted in the DRC suggested that there was a degree of stigma toward both adolescent and unmarried adult mothers For instance, community members expressed a fear of losing social status or being disliked if they associated with single mothers, perceiving out-of-wedlock childbirth to be immoral and regarding single mothers as having a low value and contribution to society [14].

In cases of single mother parenting, mothers often struggle to be the principal household provider of income and resources, in addition to sustaining the same degree of attention towards monitoring and supervising their children [15]. By measuring household wealth indexes in Congo Brazzaville, Gabon, Namibia, and Swaziland, Odimegwu et al. found that single mothers were more likely to live in poor households [16]. Moreover, single mothers were more likely to be unemployed or employed in low-income positions in sales, manual labour, and/or agriculture [16]. Similarly, in South Africa, single mothers were more likely to be unemployed and/or unable to leave their children to work [17]. In many cases, under- or unemployment is related to lack of educational opportunities, with less than 1% of single mothers in Cameroon, the DRC, and Nigeria having tertiary education [6].

Research has documented inconsistencies in the amount of paternal support available for single mothers in sub-Saharan Africa. While some fathers may continue to support their children following a family breakup, others may not acknowledge their biological children because they have become the primary support to children in new relationships [7]. Conversely, fathers may not provide financial support due to economic hardship or because they are unwilling to do so [15].

Varying levels of external support have been reported for single mothers. Paternal death is often associated with significant household income losses, and financial hardship may be the greatest among widows [18,19]. In sub-Saharan Africa, children of widows are more likely to access economic resources if the mother inherited assets from their late father. However, this often does not happen [6]. In the case of separation, there is also little legal protection for single mothers. For instance, Mokomane found that in cohabitation households, women had no legal protection for inheritance, property, or maintenance rights [20].

### 1.2. Single Motherhood and Adverse Child Health Outcomes in Sub-Saharan Africa

Emerging evidence has indicated a strong link between single motherhood and adverse child health and developmental outcomes in sub-Saharan Africa. Given high levels of poverty and gender gaps in education and income, single mothers and their children are at increased risk for malnutrition and child mortality [6]. For example, Omariba and Boyle determined that across 22 sub-Saharan African countries, the risk of mortality for children in single mother households was 16.3% greater than those with mothers in monogamous union households [8]. In addition, single motherhood, either as a result of premarital childbirth or divorce, was found to increase the risk of under-5 mortality [5].

Recently it was also demonstrated that children of never married, divorced, or separated mothers in the DRC and Cameroon had a 1.79 times increased risk of having stunted growth, which is associated with poor cognition, decreased educational performance and economic status, as well as poor health in adulthood and increased risk of mortality [6]. Family structure may also relate to the educational attainment of children, acting as a precursor for greater opportunities in life and prosperous health. In one study, children from two-parent households were 1.4 times more likely to be in the right grade level for age in comparison to children from one-parent families [21]. Furthermore, children in female-headed households were at a 1.3 times increased risk of dropping out of school [22].

In the absence of a father, children may feel neglected and abandoned in terms of emotional or economical support and they may experience additional life distress and trauma [10]. Children in single mother households may also be treated differently in their maternal homes and be predisposed to abuse by caregivers outside of the home [10], potentially leading to adverse health and developmental-related consequences.

### 1.3. Purpose of the Study

A majority of children in sub-Saharan Africa will be parented by a single mother at some point in their lives [4,5,23]. With the pervasive poverty in the DRC, many families face economic hardship. However, existing literature suggests that single mothers often face unique challenges such as even fewer household resources, underemployment, low self-esteem, and diminished quality of life [24]. Additionally, in conflict settings such as the DRC, experiences of trauma and displacement often result in heightened parental stress, as well as social and economic instability [25]. With existing evidence documenting a link between single motherhood and child physical and psychological health consequences in sub-Saharan Africa [5,8,10], it is important to understand how single mother parenting relates to child health and development. However, there is limited research on this topic in the DRC.

Our study addressed the following research question: what are the perceptions and experiences of single mother parents in the DRC, including challenges faced and adaptations made while parenting in situations of adversity. More specifically, we investigated: (1) circumstances leading to single motherhood, (2) the challenges that single mothers experience and adaptations made, (3) perceptions and stigma relating to single motherhood in the community, and (4) programs and services that could benefit single mothers and their families in the DRC. In amplifying the voices of single mothers, this analysis intends to inform strategies to empower and support single mothers in the DRC.

## 2. Materials and Methods

### 2.1. Study Design and Recruitment

Data were collected through a larger project, ‘Parenting in Adversity: An International Comparative Study’, within the Queen Elizabeth Scholars (QES) network for maternal and child health equity. The cross-sectional, mixed methods research was conducted in Canada, the DRC, Mongolia, and Thailand in 2019. The original research explored strategies and adaptations that parents use when raising children in situations of adversity, including but not limited to poverty, forced displacement, armed conflict, having a disability, or threats based on race, gender, ethnicity, or sexual orientation. Within the context of this broader research project, the current analysis focuses specifically on single parenting in the DRC, where there was a disproportionate number of single mothers (263 out of 568 completed interviews). Cognitive Edge’s SenseMaker was used to simultaneously collect qualitative and quantitative data. Interviews were conducted at four locations in the Goma area of North Kivu Province in the eastern region of the DRC: Sake, Lac Vert, Mugunga, and Nyiragongo. Prospective participants were approached for participation in public locations including market areas, street vendors, public transportation stops/depots, and their homes. Participants were required to be parents or caregivers, defined as living with and raising a child, or having lived with and raised a child in the past. No prior relationship existed between researchers and study participants.

### 2.2. Survey

SenseMaker is a narrative-capture tool that collects anonymous micronarratives, or stories, shared by study participants on a topic of interest. The story prompts were intended to trigger recall of a past parenting experience. Each narrative prompt was open and allowed for positive or negative stories to be shared, depending on the choice of the narrator. In response to a story prompt about parenting (see Appendix A: Table A1), participants shared micronarratives that were audio-recorded. Participants were then invited to interpret their micronarratives by plotting their perspectives between two (dyads) or three (triads) possible responses. Plotted data points were quantified by SenseMaker, providing statistical data for each micronarrative.

The SenseMaker survey was developed by an experienced research team and a SenseMaker consultant. The survey was written in English, then translated into Swahili, and independently back-translated to ensure accuracy and to resolve translation discrepancies. Standard multiple-choice questions were used to gather demographic data and additional information about the micronarratives, such as who the child was, how the participant felt about the experience, and the degree of adversity faced by the family. The types of household adversity asked about in the survey were based on the original adverse childhood experiences research by Felitti et al. [26]. However, since the questions were asked differently from the original research, we have intentionally avoided using the term adverse childhood experiences or ACEs, preferring instead to use household adversity as a broader concept. Full survey questions are available in Appendix A (Table A1).

### 2.3. Study Implementation

Twelve Congolese research assistants who were students or graduates of Goma-based Université Libre des Pays des Grands Lacs (ULPGL) facilitated data collection. The team completed a four-day training immediately prior to data collection, covering research ethical principles, data collection procedures, an introduction to SenseMaker methodology, sampling, and data entry, as well as reporting of adverse events, and making service referrals. Interviews were conducted face-to-face in Swahili using the SenseMaker application (Cognitive Edge, Singapore) on handheld tablets. In cases where participants had limited literacy skills, the research assistant read the survey aloud and facilitated its completion with the participants. Recorded micronarratives were later transcribed and translated into English by a professional Congolese translator.

### 2.4. Ethical Considerations

The Queen’s University Health Sciences and Affiliated Teaching Hospitals Research Ethics Board (protocol #6025181) and the Congolese National Committee of Health Ethics approved this study. Participants were assured confidentiality; no identifying information was collected, and all data were uploaded to a secure server. Informed consent was obtained by participants tapping a consent box on the tablet. The consent form could be read aloud, and verbal consent could be provided if requested by participants. Participants were given the opportunity to ask questions and address any concerns. In recognition of their time (~15 min), each participant was offered the equivalent of $1 USD.

### 2.5. Analysis

**Figure 1:**
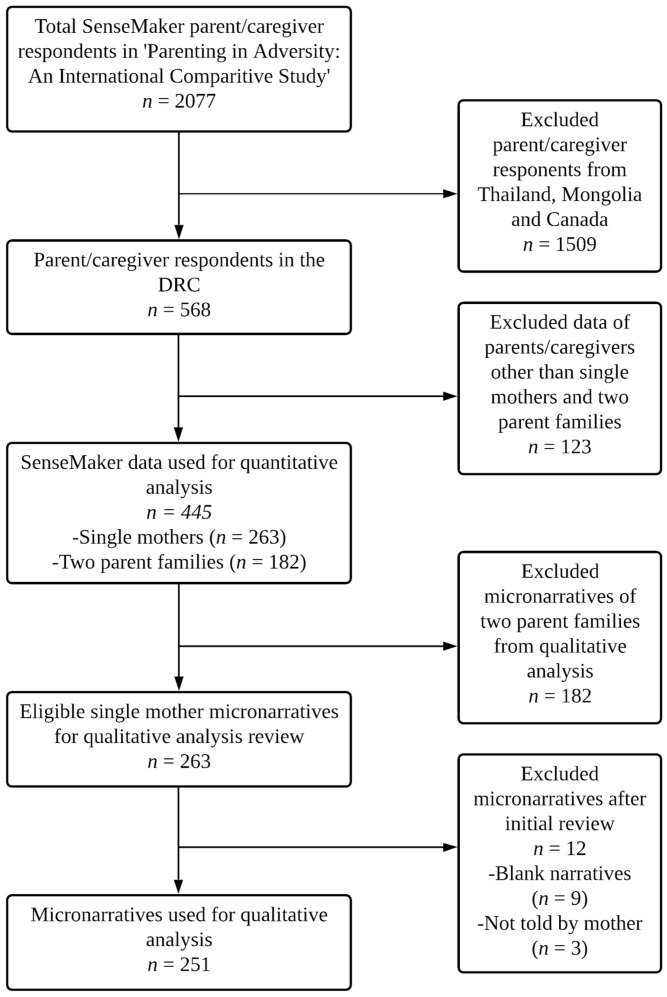
Qualitative and quantitative data selection and analysis procedure.

From the larger dataset of all Congolese respondents (*n* = 568), quantitative analysis involved a comparison of SenseMaker data from single mother (*n* = 263) and two-parent family (*n* = 182) respondents. Responses of caregivers who did not identify as a single mother or member of a two-parent family were excluded from the analysis. Single mother micronarratives (*n* = 263) were selected for qualitative analysis. Twelve micronarratives were removed during the initial review (no narrative recorded *n* = 9 and respondents were a single father *n* = 3), leaving a final sample of 251 for qualitative analysis. See Figure 1 for an overview of the participant selection and analysis procedure.

### 2.6. Quantitative Analysis

SenseMaker’s quantitative data were generated from participants’ interpretation of their shared experiences in the form of plotted perspectives between two (dyad) or three (triad) options, in addition to the multiple-choice questions that collected demographic and contextualizing data. SenseMaker plots were visualized in Tableau V.2020.4 (Tableau, Seattle, WA, USA) to identify response patterns between single mother and two-parent respondents. Plots that appeared to have visually different response patterns between the two subgroups were selected for statistical analysis.

Dyad data were generated into histograms with 11 equal bars, which represent a frequency distribution of responses between two extremes. In SPSS (IBM SPSS Statistics V.24.0.0.0, IBM, Armonk, NY, USA), the Kruskal–Wallis H test with a chi-squared test statistic was used to determine if bar areas were statistically different between subgroups [27,28]. Differences were declared statistically significant with a *p*-value < 0.05 and the distribution of responses were re-plotted as violin plots to illustrate the different response patterns, with an asterisk indicating the overall mean for each sub-group.

Quantitative analysis of triad data used R scripts (R V.3.4.0, R Foundation for Statistical Computing, Vienna, Austria) to generate geometric means with 95% confidence intervals, represented as confidence ellipses, for each subgroup [29]. Statistical significance was determined if the 95% confidence ellipses did not overlap between subgroups.

For the multiple-choice questions, chi square tests were conducted to examine differences in responses of single mothers versus two-parent families. Findings are presented with corresponding odds ratios and 95% confidence intervals. Odds ratios were generated with an online calculator, MedCalc (https://www.medcalc.org/calc/odds_ratio.php (accessed on 16 August 2021)). A *p*-value < 0.05 was considered statistically significant. The survey used the term “both parents” and for the purposes of this analysis, we refer to two-parent families versus single mother families. However, we did not collect data on what was meant by “both parents” and therefore cannot confirm that it referred to the biological mother and father.

### 2.7. Qualitative Analysis

After familiarization with applicable literature and a thorough review of single mother micronarrative transcripts, MC and SB conducted a thematic analysis using an adaptation of Braun and Clarke’s approach [30]. In our adaptation, a thematic codebook was co-developed and using Dedoose (V 8.3.43) (Dedoose, Los Angeles, CA, USA), MC coded each micronarrative line-by-line. SB co-coded 51/251 micronarratives (20%). Following initial coding, MC reviewed first-level codes and organized the codes into larger thematic categories. The themes were not mutually exclusive; micronarratives were often placed into more than one thematic category, reflecting the potential range of ideas, feelings, or experiences involved in a single micronarrative. MC and SB engaged in dialogue throughout the qualitative analysis, communicating through both written memos and conversation to assess coding questions, biases, and discrepancies. Quotes from micronarratives highlighting diverse perceptions in each category were selected to illustrate relevant themes. Using a mixed-methods approach, the quantitative findings informed the qualitative codebook for thematic analysis and the identified qualitative themes were subsequently mapped onto complementary quantitative findings to increase depth and understanding.

## 3. Results

Demographics and micronarrative characteristics are provided in Table 1 disaggregated by single mothers and two-parent families. A majority of both single mother participants (68.82%) and two-parent family respondents (81.87%) were aged 18–44. The highest number of child protagonists were aged between 12–18 and there was a slight preponderance of female children. Single mother participants and two-parent family participants both predominantly indicated poorer relative wealth compared to others in their communities.

### 3.1. Thematic Categories

**Figure 2:**
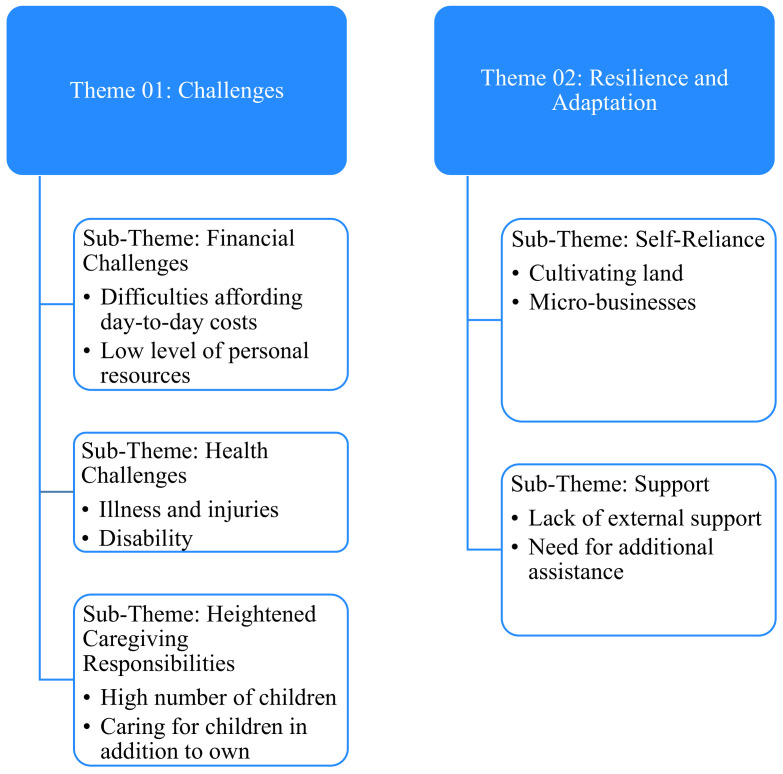
Qualitative themes and sub-themes.

Two thematic categories: (1) challenges plus (2) resilience and adaptation emerged from our analysis. Figure 2 provides a summary of sub-themes discussed under each overarching thematic category.

### 3.2. Challenges

Most single mother participants highlighted challenges associated with raising children in the DRC, using language such as “suffering” and phrases like “this is the life I am leading” or “I am leading a difficult life” to describe hardships (ID 2038, ID 2134). Quantitative data supported this thematic finding with single mothers being 1.83 times more likely to indicate that their micronarratives were negative (OR = 1.83, 95% CI 1.13–2.95) compared to two-parent family respondents, reflecting feelings such as hopelessness, frustration, worry, stress, sadness, and disappointment. Three prominent sub-themes related to challenges faced by single mother families were identified: (1.1) financial challenges, (1.2) health challenges, and (1.3) heightened caregiving responsibilities.

#### 3.2.1. Financial Challenges

Poverty was a recurrent theme and many mothers experienced problems related to raising children in impoverished conditions, including affording day-to-day costs, and having a low level of personal resources. Single mother participants often attributed their economic struggles at least partially to the fact that they were single parents, and their children were without the financial and material support of the father. While poverty is widespread, and in many cases extreme, in the DRC, financial challenges were often exacerbated for single mother participants.

##### Difficulties Affording Day-to-Day Costs

Difficulties affording day-to-day costs were categorized based on the specific financial burden experienced by the single mothers (ex. food, healthcare, children’s schooling). However, most micronarratives mentioned several overlapping economic challenges and the following narratives illustrate how these difficulties were central stressors in the lives of participants.

Single mothers often portrayed themselves as helpless against poverty, alluding to physical and emotional exhaustion from constant financial challenges. Difficulties affording food and feeding children adequately was the most prominent concern among single mothers facing financial struggles, as in the narrative below:

*“I am a widow. I live here with my children. We are suffering so much. In reality, because of poverty, we miss what to eat. As you see us here since the morning nobody has eaten. It means things are very hard. I don’t know what to do because when the sun sets, we go to bed, but we don’t always know if it will rise again for us. We are so poor that we sometimes assume we will die when we go to bed. In fact, we are suffering a lot.”*

*(ID 1923)*

Secondary concerns for mothers included affording their child/children’s education or related costs such as uniforms and books. Oftentimes, mothers described children being unable to attend school due to financial barriers:

*“None of my children go to school… The first one is 8 years old. I pray that people help us pay their school fees... My husband left 3 years ago.”*

*(ID 1771)*

Many single mothers expressed a sense of vulnerability in providing for day-to-day costs associated with parenting. In the following example, the mother saved part of her daily earnings and attempted to negotiate with teachers to send her children to school, but it wasn’t always sufficient:

*“They are fatherless. I live with them alone. The first one is 13 years old, but because of poverty, he is now in 3rd primary school. I work for CDF 2000 per day. I use CDF1500 and save CDF 500. I save it to pay school fees. I negotiate with teachers after I saved a given amount of money. Sometimes the teachers don’t put up with me, so they chase them [the children]. And in that case, they stay at home. All of this is because of poverty”*

*(ID 2134).*

##### Low Level of Personal Resources

In addition to direct financial barriers, single mothers often described having low or insufficient levels of personal resources, including land, material goods, and/or stable housing. For example, one mother noted losing land to cultivate due to conflict-related displacement, which resulted in exacerbated financial struggles including having to rent a home:

*“We displaced from there because of war. We lost all of the production within our fields. We are suffering here because we don’t have land to cultivate in. As a result, we are renting houses here.”*

*(ID 1875)*

Mothers who experienced loss of resources, rather than a low level of resources to begin with, may have experienced particularly increased barriers as single parents. Renting housing or relying on external employment opportunities tended to increase uncertainty. For instance, the following mother described facing expulsion from their home:

*“My husband is a soldier. He left me with 7 children, none of whom go to school. I live with them right here... I really need support because I am even renting a house where they are expelling me. This is the kind of life I am leading.”*

*(ID 1842)*

#### 3.2.2. Health Challenges

Health complications, including illness, injuries, and health-related disability, were frequently mentioned in the single mothers’ micronarratives. Mothers primarily spoke to their child/children’s health status rather than their own. The majority of complications described were physical, although mental health struggles were noted by some mothers. While the research team recognizes the value of framing disability as a human rights issue rather than a health issue, the participants themselves primarily discussed disability as being health related. For this reason and to be true to the participants’ perspective, we have chosen to present disability under health for the purpose of this analysis.

##### Illness and Injuries

The following mother gave birth to a sick child and had limited means of caring for the child’s health complications due to financial constraints, again highlighting poverty as a central theme in the lives of Congolese single mother families. Micronarratives such as this were prominent with lack of money being a significant barrier to accessing adequate care and often resulting in adverse health outcomes for the child or children:

*“Since I gave birth to X, she is sickly. I have had her treated, but nothing is improving because I don’t have money. She doesn’t even eat well. She has changed completely. I don’t know what is going on in her body, for her skin is changing completely since I gave birth to her.”*

*(ID 1690)*

Other mothers mentioned specific illnesses, such as Kwashiorkor, a severe form of malnutrition resulting from insufficient dietary protein or lack of essential nutrients. Despite the following mother seeking help, the child had ongoing adverse health consequences due to inadequate nutrition:

*“That child made me suffer because his father died while he was still young…I have struggled a lot with him…When he was a baby, he suffered from Kwashiorkor. I took him to the place where he could eat porridge they give to malnourished children. He is 18 years old, but he is not strong enough because of what he endured…”*

*(ID 1851)*

Single mothers repeatedly expressed emotional hardship when not able to secure appropriate healthcare for their children, claiming they “*missed what to do*” *(ID 1925, ID 1946, ID 2085…)*. Child mortality was mentioned at times, either as a possibility given concurrent health issues, or in cases of child deaths due to health conditions or injuries. Many mothers expressed a sense of their children’s health and life largely being outside of their control, as described in the narrative below:

*“When you have many children in your household, you face a lot of problems. When they fall sick, you become overwhelmed with sorrows because of lack of means to help you out. One of my children fell seriously sick to the extent that we were worried about his fate. Happily, he made it, but in other situations we can even lose a person or a child because of lack of money.”*

*(ID 1748)*

##### Disability

Single mothers were 1.7 times more likely to report that they were parenting a child with a disability compared to two-parent families (OR = 1.7, 95% CI 1.13–2.55). Of all single mother respondents, 52 (19.85%) and 32 (12.21%) indicated that someone in their micronarratives were living with physical or psychosocial disability, respectively. Within the single mother narratives, mothers often explained how health complications and sometimes improper, unsuccessful or lack of medical care contributed to a more long-term physical disability. Narratives that fit into this criterion were included under the larger ‘health struggles’ theme, however, disability was occasionally mentioned in the absence of a health component. A mother shared an experience of disability early in the child’s life despite seeking medical care:

*“That child was born well, but when he turned 7 months old, he started falling sick, he fainted down, and one side become unfunctional. They took him to hospital. That situation was unsuccessful, that is why he is a disabled child.”*

*(ID 1673)*

Other mothers explained that their child was born with a disability, unrelated to a pre-existing health condition, such as in the following narrative:

*“I was married at the age of 13. I suffered a lot while I was pregnant. When I delivered the child, he was born with disabilities. The child has made me suffer up to this time. My husband died. I started searching here and there, as I was left alone to take care of the child.”*

*(ID 2004)*

This narrative emphasizes the emotional toll and stress that single mothers may experience when raising a child with a disability on their own, evident by her saying, “the child has made me suffer up to this time”.

#### 3.2.3. Heightened Caregiving Responsibilities

While several parenting difficulties were highlighted in single mother micronarratives, two prominent themes were (a) parenting other children in addition to their own, and (b) parenting a high number of children, defined as >6 children in our analysis.

##### Caring for Other Children in Addition to Own

Single mothers parenting high numbers of children was partially attributable to caring for orphaned children. For example, the story below emphasizes caring for orphans as the mother’s own. The micronarrative also implied that parenting a high number of children contributes to financial struggles and a reduced parenting capacity to provide for the children’s needs:

*“I am a widow living with my 10 children and 2 other orphans. In total, I have 12 children. We don’t have money to rent a house. We are living in a house to be the watch people for the house. Since I don’t have means, my children don’t go to school, and I don’t have means to feed them, send them to school, and clothe them.”*

*(ID 2076)*

Mothers also specified caring for their daughter’s children in the event of premarital pregnancy, father abandonment, or a failed marriage causing the daughters to return home. Highlighted below is a story involving sexual violence, resulting in the daughter’s pregnancy and subsequent reliance on the mother for support. The mother explained the negative emotional impact for her and her daughter:

*“I am suffering too much. I was married to a man who died and left children. I have a daughter of mine who has 2 children. She was raped... She became pregnant. She gave birth to a child whose legs are dangled... We are suffering so much. My daughter is suffering too.”*

*(ID 1725)*

In this example, caregiving responsibilities were likely further heightened if the participant’s grandchild had a disability as implied by the description of ‘dangled legs’.

##### High Number of Children

The narrative below showcases the added difficulty of parenting a high number of children, reflective in the mother not being able to send all her children to school like she previously had:

*“I am a mother to 8 children. My husband died while I was pregnant of the last born. At that time, I was sending 5 children to school, but for the time being, I only pay for 1 child, so others are staying at home.”*

*(ID 1886)*

Single mother micronarratives were 2.1 times more likely to detail household adversity (OR 2.1, 95% CI 1.4061–3.1521), including physical and emotional neglect, substance use, and mental health/suicide, compared to two-parent family respondents. Thus, the quantitative data supports the exacerbated parenting difficulties identified in the qualitative analysis. The odds ratio calculation excluded ‘loss of a parent’ as an adverse experience since this would have been naturally higher in single mother households. Similarly, the calculation also excluded ‘child is an orphan’ since ‘orphan’ is sometimes used in African cultures to refer to a child who has lost one parent (paternal orphan or maternal orphan).

### 3.3. Resilience and Adaptation

**Figure 3:**
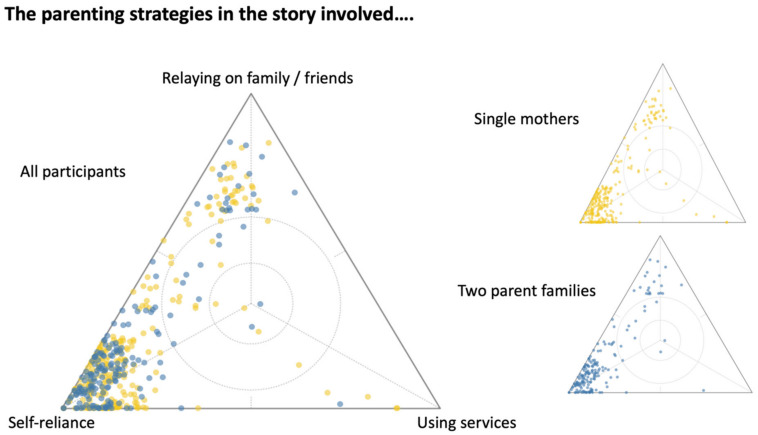
Triad showcasing single mother and two-parent family’s perspectives on parenting strategies.

As seen in Figure 3, in the triad asking about parenting strategies, most participants (both single mothers as well as two parent families) responded heavily towards the ‘self-reliance’ option.

Our qualitative analysis also indicated that single mothers relied primarily on themselves to earn an income and support their children to the best of their abilities. The majority of mothers were unemployed or worked in precarious, informal employment, depicting a self-reliant coping strategy. Mothers cultivated land, sold items, and took out loans to cover parenting expenses; they often described taking on these tasks as the only available means of providing food, paying for school fees, or affording healthcare. Two qualitative sub-themes are discussed: (2.1) self-reliance and (2.2) support.

#### 3.3.1. Self-Reliance

In the context of financial challenges and other adverse circumstances, self-reliance was prominent among the single mothers. Many participants prioritized their children’s needs above their own and exhausted all opportunities to afford childrearing costs such food, education, and healthcare. Two aspects of self-reliance are illustrated within this thematic category: cultivating land and micro-businesses.

##### Cultivating Land

A number of single mothers engaged in agriculture to earn an income. The following mother described cultivating land as “carrying a burden” to feed and clothe her children, indicating that it was demanding work:

*“I have difficulty finding what to feed my children. I am suffering so much. My children don’t even have what to wear as we don’t have things to do here. I am cultivating beans on this rock, but cows and goats are sent to eat our production, that is why raising these children is a very serious issue for me… If I don’t carry burdens, my children can’t find what to eat and what to put on their bodies.”*

*(ID 2010)*

Some mothers added that working in the fields wasn’t sufficient to meet their families’ needs, leading to an endless cycle of informal work for little pay. In the subsequent story, the mother indicated that cultivating was the best and likely only viable option for feeding their child:

*“I go to the fields to cultivate for people in exchange for money so as to feed my child. The father of the child doesn’t care about him. I do my best to find something for him. I don’t know what to do; maybe I will be going to the fields where they give CDF 2000, so I will be saving a half and consume a half. I can’t start up an economic activity with that amount.”*

*(ID 1806)*

##### Micro-Businesses

Many single mothers relied on micro-business endeavours or small jobs, including gathering and selling items such as charcoal and firewood, to support their children. In the following narrative, a mother indicated that she was “used to searching” for items to sell, further supporting the reality of unpredictable employment among single mothers:

*“I raised 6 children without living with their father. It was in Masisi. I work menial jobs in order to feed them and provide them with health care. Nobody supports me. I came here because of poverty. I am currently living with 3 young children here. I am used to searching for firewood to feed my children.”*

*(ID 2007)*

Some single mothers indicated that they received loans to help fund start-up businesses, such as in the following narrative:

*“For the time being, my child is now in 3rd form primary school. I don’t have any good occupation to raise him properly in good conditions. I take out loans that I pay with interest at Kituku in order to do small business. I get something that I use to pay school fees and feed him.”*

*(ID 1685)*

In many scenarios, micro-business activities provided insufficient income to support all childrearing expenses in most single mother micronarratives. In the after mentioned narrative, the mother covered healthcare and food-related expenses with the income generated. However, as explained in the next narrative, when a mother dedicated a portion of micro-business earnings toward their children’s schooling, she could not support adequate nutrition. The mother described being able to make one meal of potatoes that would have to suffice for the day:

*Life is very difficult. My only job is selling firewood. If I get a small amount from a sale, I take that to school to pay their school fees. They eat difficultly. For example, I have CDF500 I buy potatoes that I cook for them in the morning, and when they come from school, they take the remaining.*

*(ID 1679)*

#### 3.3.2. Support

In our analysis, accessing support was defined as reliance on others or use of services. In general, support was an overarching, noteworthy theme that was mentioned in many single mother micronarratives; whether discussing if supports were available or not, who or what the mother relied upon for support, or what support would be beneficial.

##### Lack of External Support

The majority of single mothers described a lack of available support, using phrases such as “nobody helps me” (ID 1863), “I have nobody to help me. I don’t know where I have to knock for support” (ID 2010), and “I play two roles at once—the father and the mother. Raising these 6 children is very difficult since I don’t have support” (ID 1943). Many mothers did not mention whether systematic supports and services existed or not.

**Figure 4:**
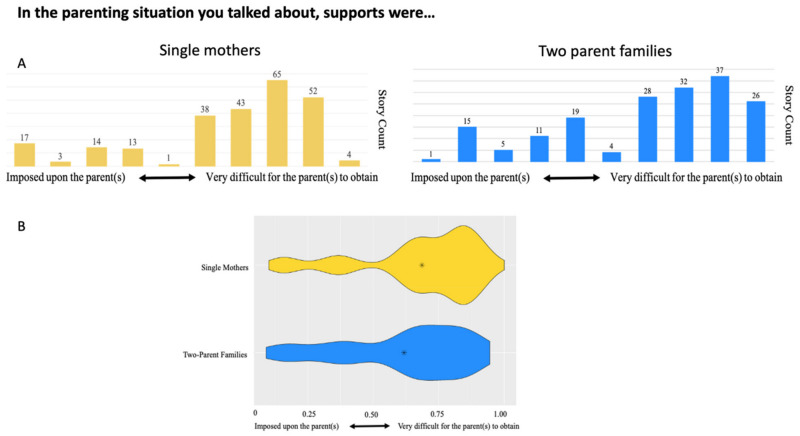
Dyad story counts (**A**) and violin plots (**B**) illustrating single mother and two-parent family responses to whether supports were imposed upon the parent(s) or very difficult for the parent(s) to obtain. Single mothers were more likely to respond that supports were difficult to obtain (*p* = 0.001). The asterisk indicates the mean response for each subgroup.

Our thematic analysis suggested a relationship between lack of support for single mothers and the use of self-reliance as a primary parenting strategy. This determination was made primarily due to a high number of micronarratives mentioning both support and self-reliance together, and from the language single mothers used to describe the challenges they faced. The quantitative data also supported these qualitative findings, with single mothers being more likely to indicate that supports were difficult to access (*p* = 0.001*)*. As shown in Figure 4A,B, in comparison to two-parent families, single mother responses trended more towards supports being ‘very difficult for the parent(s) to obtain’, with a higher mean (indicated by the asterisk) and an overall distribution that was ‘wider’ towards the difficult to obtain extreme on the dyad.

##### Need for Additional Assistance

Many mothers indicated a need for support, coupled with a drive to improve life circumstances for themselves and their children if supports were available. Support-seeking behaviours were most frequently rooted in motivation to provide educational opportunities for their child(ren). As one mother described:

*“My children study difficultly. When they chase them from school, I don’t feel okay. They are fatherless. I feel worried about that. If I get someone to help me, my children can study. I didn’t have a chance of going to school, so I say that the only legacy I can leave to my children is to make sure I send them to school.”*

*(ID 2014)*

Other mothers requested support to become financially independent or to have greater control of their finances, most likely to escape from inconsistent or unpredictable employment. In the following narrative the mother sought start-up capital so she could begin her own financial endeavours rather than assist a relative in theirs:

*“…My husband has left me again. I am only living with my uncle’s wife whom I help her deal with her business... My request is to have my own start-up capital to be self-reliant financially.”*

*(ID 1955)*

This detailed micronarrative reinforces the concept of single mothers in the DRC being driven to improve their situations if provided with the necessary support and opportunities. Though single mothers demonstrated resiliency and adaptations while parenting in adversity, overall qualitative and quantitative findings suggested that additional support is critical in helping single mothers reach their full potential as independent caregivers.

## 4. Discussion

In the context of a conflict-affected population in eastern DRC, this study explores the phenomenon of single mother parenting in situations of adversity. Through micronarratives and the single mothers’ interpretations of those experiences, the findings provide insight into the degree and types of adversity experienced among single mother families. Two overarching qualitative themes, ‘challenges’ as well as ‘resilience and adaptation’ emerged from the data. Overall, identified themes suggest that single mothers in the DRC are struggling, due to ongoing poverty, to raise their children as independent caregivers amid gender inequalities and lack of social services/external supports. Economic deprivation was the strongest contributing factor to adverse child health and developmental consequences, often resulting in an inability to meet children’s educational, nutritional, and healthcare needs. Many single mothers highlighted their financial struggles in the context of being a single parent, implying that without support from the children’s father, the poverty experienced by single mother families is possibly exacerbated beyond that which would be expected in the broader community. Household adversity was more commonly documented in single mother micronarratives compared to two-parent micronarratives, potentially due to exacerbated challenges among single mothers to provide for their child(ren). Single mothers documented emotional distress because of their parenting circumstances, and with a general lack of support available, they relied primarily on their own resourcefulness to provide for their children despite the adversities faced.

The micronarratives revealed many barriers to accessing employment and resources, which is consistent with prior research showing that less educated single mothers in Africa faced heightened barriers to accessing resources and income [6,13,31]. We found that these barriers were exacerbated by discriminatory gender norms surrounding property and land ownership rights in the DRC, which have also been well-documented [2,32]. For instance, legally married women in the DRC had no right to access property or lands and were unable to sign legal contracts without the approval of their husbands prior to the family code being recently modified. Though marital authorization is now repealed, in cases of father abandonment, husbands may still take all resources and assets and leave a woman with little means for economic survival [2,33]. Moreover, in DRC land use and distribution are largely regulated by local customary chiefs. These positions are almost exclusively reserved for men and discrimination against women remains strong [32], creating obstacles for single mothers to obtain or retain resources. Our findings call for further investigation into whether recent modifications to historically discriminatory laws and policies have resulted in changes to women’s experiences and allowed for greater economic freedom.

Health-related challenges such as illnesses, injuries, and malnutrition-related diseases were prominent in our analysis and mothers often could not afford adequate care for their child(ren). Mothers were statistically more likely to report parenting a child with a disability compared to two-parent family respondents. In our qualitative analysis, we presented disability as a health-related subtheme since this is how it was discussed by most single mother participants. However, the authors recognize that disability is not always a health-related challenge. Though it was difficult to determine the underlying cause of disabilities, unaffordable healthcare contributing to potentially preventable long-term disability was raised in several micronarratives. Furthermore, with the 2013–2014 DRC Demographic and Health Survey data indicating that approximately 76% of women face barriers accessing maternal health care [34], limited access to pre-natal care and supported deliveries could contribute to children being born with disabilities. However, further investigation is needed to examine these relationships.

Heightened parenting responsibility was described by single mothers, including parenting a high number of children and caring for other children in addition to their biological offspring, with the later contributing to the former in certain situations. Mothers described financial as well as emotional consequences of caring for additional children or high numbers of children, most often due to increased parenting demands and limited capacity to support the children. Contradicting the theory that extended family are commonly used for caregiving support in sub-Saharan Africa [35], relying on family for support was only sometimes mentioned in our micronarratives, and mainly in the case of single mothers caring for their daughters’ children. Rather, single mother participants more commonly indicated that they relied on neighbors or other community members for support, and solely for the purpose of providing the mother with employment opportunities. Some single mothers expressed poor mental and psychosocial well-being, and they were more likely to describe their micronarratives as negative compared to two-parent family respondents, illustrated by feelings of hopelessness, frustration, worry, stress, sadness, and disappointment. This finding aligns with previous literature reporting that single mother parenting was associated with maternal mental health disparities in other sub-Saharan African contexts [6,24].

While experiencing immense challenges and barriers raising children in adversity, single mothers often described self-reliance, commonly working in informal and unpredictable employment to earn a small income. Self-reliance was often rooted in survival as suggested by language used by mothers such as ‘carrying burdens’ to provide for the child(ren) to the best of their abilities. Our findings agree with previous research revealing that single mothers were more likely to be unemployed or employed in low-income positions as vendors, manual labour, and agricultural industries [16]. Like other studies in sub-Saharan Africa [3,15,36], our research also highlights that there is little support available to single mothers. Although some mothers indicated receiving a degree of support from others, most indicated receiving no assistance for their child(ren). There is little existing literature on support networks for single mothers in sub-Saharan Africa, and research rarely distinguishes between practical, emotional, and social support [37]. Our dyad data indicated that supports were difficult to access and very few single mothers responded towards ‘using services’ as a parenting strategy in the triad. Although it was difficult to determine from the data whether the issue was lack of supports and services versus accessibility, historically the DRC has not had strong social service provision [38].

### 4.1. Implications for Research and Practice

Additional localized research should be directed towards identifying the needs of single mothers in the DRC. It is also important to understand how the recently changed Congolese legislation regarding women’s ability to own property and to sign legal contracts may or may not impact the ability of single mothers to meet the needs of their families. These data can help to better inform policies and programs to support single mothers raising children in adversity such as conflict, forced displacement and extreme poverty. Through the Sustainable Development Goals, the United Nations International Children’s Emergency Fund (UNICEF) hopes to “grant every child a fair chance in life, ensuring their health, safety, education, and empowerment” [39]. However, in the DRC, social services and support programs aimed at improving child health and development are significantly lacking [40]. In developing interventions, practices to date have predominantly focused on the relationship between income, women’s education, and environmental factors with child health outcomes [41]. Although less attention has been given to understanding the influence of single mother parenting, poverty was prominently featured in our findings, adding support for the provision of educational and economic opportunities for single mothers.

Microfinance programs, including micro-credit, micro-savings, and micro-insurance may alleviate poverty by allowing marginalized individuals to receive equitable access to financial services [42]. Such programs have been credited with providing direct economic benefits through essential start-up loans, improving savings, and acquisition of assets. Moreover, microfinance programs are believed to empower women to gain financial skills, own bank accounts, and improve employment mobility, in addition to contributing to social cohesion, better health and education, as well as job creation [42]. Productive entrepreneurship through microfinance programs could help single mothers escape low-paying and inconsistent work in the informal employment sector, which was a barrier to mothers’ financial security in our study. However, the efficacy of these programs in fully addressing single mothers’ needs is not well-documented in the DRC.

Independent of microfinance programs, financial barriers, rooted in gender inequity in sub-Saharan Africa, continue to limit women’s employment and earning potential [43,44,45]. Limited access to assets and resources such as land and property, as well as constrained credit access, act as barriers to women’s entrepreneurship and microenterprises [44]. Thus, policies and programs geared toward labour market gender equity should consider upstream factors of income-generating employment ventures and productivity, such as skills training, working capital, access to resources and social support. As suggested by Raniga, transformative interventions by government, non-governmental organizations, and private sectors need to fully address the social and economic exclusion experienced by low-income single mothers [46].

### 4.2. Strengths and Limitations

It is important to acknowledge the limitations of this study. First, convenience sampling was used to collect data; thus, our findings do not represent the perceptions and experiences of all single mothers in the DRC and are not generalizable. Second, SenseMaker micronarratives are shorter in comparison to traditional qualitative interviews and without probing questions, important details may not have been captured. The micronarratives were also transcribed from Swahili into English, opening the possibility of translation discrepancies and loss of language-or-culturally specific nuances. SenseMaker quantitative data from 263 single mothers were used to calculate odds ratios, however, only 251 single mother narratives were analyzed thematically due to exclusion criteria and blank narratives. The scope of this project did not allow for coding of all the two-parent micronarratives. Therefore, only single mother micronarratives were used for the qualitative analysis, and qualitative differences between single mother and two-parent micronarratives could not be identified. Further, several of the thematic concepts discussed may not be exclusive to single mothers in the DRC as poverty was a core underlying factor for many Congolese families who participated.

The study also has several notable strengths. To our knowledge, this is the first empirical study examining perceptions and experiences specific to single mother parenting in the DRC. Open-ended story prompts empowered single mothers to share micronarratives of their choosing, contributing to a breadth of rich and diverse experiences. SenseMaker’s quantitative data reduced interpretation bias since participants interpreted their own experiences and we were able to capture a large sample using the SenseMaker application. Additionally, participants were invited to partake in the study from four different communities in the greater Goma area, allowing for a more diverse sample. Our non-Congolese research team members spent a prolonged time in the field and collaborated closely with Congolese colleagues to understand cultural-specific nuances and mitigate interpretation biases. Lastly, the mixed quantitative/qualitative analysis contributed to a more comprehensive understanding of single mother parenting in the DRC.

## 5. Conclusions

Single mothers in the DRC face significant financial, health and parenting-related challenges raising children in adverse situations, which may translate into negative child health and developmental outcomes. Single mother participants had little support raising children and primarily exhibited self-reliance to cope with the challenges they faced. Our qualitative findings were supported by the quantitative results derived from participants’ interpretation of their own micronarratives: single mothers were statistically more likely than two-parent family respondents to report household adversity, to parent a child with a disability, and to perceive their micronarratives as negative. Results reinforce that in the DRC, poverty, a lack of social services and support programs, as well as gender inequality, contribute to significant hardship and societal barriers for single mothers. Additional research is needed to clarify what policies and programs could best address challenges faced by single mothers in the DRC to improve health outcomes for mothers and children.

## Figures and Tables

**Table 1:** Demographics disaggregated by single mother family (*n* = 263) versus two-parent family (*n* = 182).

Demographic	All Participants (*n* (%))	Single Mother Participants (*n* (%))	Two-Parent Family Participants (*n* (%))	* *p*-Value
Age of participant (years)				0.389
12–17	5 (1.12)	5 (1.9)	0 (0)	
18–24	72 (16.18)	50 (19.01)	22 (12.09)	
25–34	137 (30.79)	65 (24.71)	72 (39.56)	
35–44	121 (27.19)	66 (25.10)	55 (30.22)	
45–54	81 (18.20)	54 (20.53.17)	27 (14.84)	
Prefer not to say	29 (6.52)	23 (8.75)	6 (3.30)	
Total	445 (100)	263 (100)	182 (100)	
Age of child in story (years)				0.375
Less than a year	11 (2.47)	8 (3.04)	3 (1.65)	
1–5 years	92 (20.67)	53 (20.15)	39 (21.43)	
6–11 years	108 (24.27)	60 (22.81)	48 (26.37)	
12–18 years	130 (29.21)	72 (27.38)	58 (31.87)	
19 years or older	60 (13.48)	41 (15.59)	19 (10.44)	
Not sure	44 (9.89)	29 (11.03)	15 (8.24)	
Total	445 (100)	263 (100)	182 (100)	
Sex of child in the story				0.628
Female	220 (49.44)	135 (51.33)	86 (47.25)	
Male	200 (44.94)	114 (43.35)	85 (46.70)	
Other	25 (5.62)	14 (5.32)	11 (6.04)	
Total	445 (100)	263 (100)	182 (100)	
** Disability in the family				0.022
No disability	315 (66.74)	178 (67.94)	137 (72.90)	
Physical	80 (16.95)	52 (19.85)	28 (14.90)	
Psychosocial	39 (8.26)	32 (12.21)	7 (3.70)	
Intellectual	16 (3.39)	8 (3.05)	8 (4.30)	
Hearing	7 (1.48)	3 (1.15)	4 (2.13)	
Visual	8 (1.70)	6 (2.29)	2 (1.10)	
Other	7 (1.48)	5 (1.91)	2 (1.10)	
Total	472 (100)	284 (100)	188 (100)	
Relative wealth compared to other community households				0.563
Poorer	265 (59.55)	155 (58.94)	110 (60.44)	
Same money	49 (11.01)	27 (10.27)	22 (12.09)	
Wealthier	11 (2.47)	8 (3.04)	3 (1.65)	
Not sure	120 (26.97)	73 (27.76)	47 (25.82)	
Total	445 (100)	263 (100)	182 (100)	
Location of interview				0.065
Nyiragongo	180 (40.45)	93 (35.36)	87 (47.80)	
Sake	89 (20.00)	59 (22.43)	30 (16.48)	
Lac Vert	90 (20.22)	56 (20.91)	34 (18.68)	
Mugunga	86 (19.33)	55 (21.29)	31 (17.03)	
Total	445 (100)	263 (100)	182 (100)	
Degree of Adversity				0.293
None	34 (7.64)	20 (7.60)	14 (7.69)	
Very little	11 (2.47)	7 (2.66)	4 (2.20)	
Some	34 (7.64)	16 (6.08)	18 (9.89)	
A lot	152 (34.16)	86 (32.70)	66 (32.26)	
Extreme	213 (47.87)	134 (50.95)	79 (43.41)	
Not sure	1 (0.22)	0 (0)	1 (0.55)	
Total	445 (100)	263 (100)	182 (100)	
Emotional tone of story				<0.0001
Negative	331 (74.38)	214 (81.37)	117 (64.29)	
Positive	86 (19.33)	43 (16.35)	43 (23.63)	
Neutral	28 (6.29)	6 (2.28)	22 (12.09)	
Total	445 (100)	263 (100)	182 (100)	
** Household Adversity				<0.0001
No adverse experience	242 (49.49)	121 (39.93)	121 (65.05)	
Physical neglect	89 (18.20)	60 (19.80)	29 (15.59)	
Child is an orphan	56 (11.45)	49 (16.17)	7 (3.76)	
Emotional neglect	40 (8.18)	25 (8.25)	15 (8.06)	
Mental health/suicide	39 (7.98)	29 (9.57)	10 (5.38)	
Loss of parent	14 (2.86)	13 (4.29)	1 (0.537)	
Substance abuse	9 (1.84)	6 (1.98)	3 (1.61)	
Incarceration	0 (0)	0 (0)	0 (0)	
Total	489 (100)	303 (100)	186 (100)	

* Chi-squared tests ** more than one response allowed and therefore sum exceeds total *n* of 445. Statistically significant results are highlighted in **bold** font.

## Data Availability

The data presented in this study are available on request from the corresponding author.

## Abbreviations

DRC, Democratic Republic of Congo

## Appendix A

**Table A1:** Survey Instrument.

Micronarrative Prompts	
Think of an example of successful or unsuccessful child rearing in this community. Describe an example for a specific child	Micronarrative recorded by participant
Think about what makes it easier or more difficult to raise a child in this community. Describe a specific parenting situation that illustrates this for a particular child	Micronarrative recorded by participant
Think of a time of when you were proud of or disappointed in your parenting or the parenting of someone you know. Tell us that story as it relates to a specific child.	Micronarrative recorded by participant
Dyads	
In the shared story, do you think the child was…	(1) Overparented (2) Underparented or a combination in-between the two extremes
In the parenting situation you talked about, supports were…	(1) Imposed upon the parent (2) Very difficult for the parent to obtain or a combination in-between the two extremes
Thinking of the story you shared, was time focused…	(1) On the present (2) On the future or a combination in-between the two extremes
Triads	
The parenting strategies in the story involved…	(1) Relying on family/friends (2) Using services (3) Self-reliance
What motivated the parenting behaviour in the story?	(1) Needs of the present (2) Cultural expectations (3) Child wellbeing
In the story shared, priority was placed on…	(1) Child’s needs (2) Parent’s needs (3) Family’s needs
What external factors primarily influenced parenting in the story?	(1) Availability of resources (2) Safety (3) Cultural norms
If parenting in the story was successful, what would be the most important outcome for the child when they grow up?	(1) Happiness (2) Financial stability (3) Having own family
Stones	
In the story shared, how effective was each of the following: Physical discipline Withholding things Showing affection Reasoning Controlling the child’s activities Yelling Shaming Rewards Ignoring or Neglect	X-axis = Effective for short term: little → a lot Y-axis = Effective for the long term: little → a lot
How helpful would each of the following be to support the parenting of the child in your story? Access to education Health services Child welfare services Financial resources Parenting instruction Women’s programs Men’s programs	X-axis = For child: little → a lot Y-axis = For parent(s): little → a lot
Multiple Choice Questions About the Micronarrative	
Who was the child in the story?	(1) My child (2) A child in my extended family (3) A child in my community (4) A child I heard or read about (5) Prefer not to say
How do you feel about this story?	(1) Negative (2) Neutral (3) Positive (4) Strongly positive
What degree of adversity was the family in the story facing?	(1) None (2) Very little (3) Some (4) A lot (5) Extreme (6) Not sure
How does this story make you feel (choose up to 3)?	(1) Afraid (2) Angry (3) Ashamed (4) Disappointed (5) Embarrassed (6) Frustrated (7) Happy (8) Helpless (9) Hopeful (10) Relieved (11) Sad (12) Worried (13) Not sure
What was the age of the child in the story?	(1) Less than a year (2) 1–5 years (3) 6–11 years (4) 12 to 18 years (5) 19 or older (6) Not sure
What is the sex of the child in the story?	(1) Male (2) Female (3) Other
What is the age of the mother in the story?	(1) Under 18 (2) 19–40 years (3) Over 40 years (4) Not sure (5) No mother in story
What is the age of the father in the story?	(1) Under 18 (2) 19–40 years (3) Over 40 years (4) Not sure (5) No father in story
Relative to the wealth level of other households in the community, is the family in your story...	(1) Wealthier than most in the community (2) Had the same money as most in their community (3) Was poorer than most in their community (4) Not sure
Is the family in the story from any of the following groups? (choose all that apply)	(1) Ethnic minority (2) Indigenous (3) Refugee (4) Internally displaced (5) Economic migrant (6) Conflict-affected (7) Disaster-affected (8) Poverty (9) Child with a disability (10) Parent with a disability (11) Person living with a physical or mental health condition (12) Unsure (13) None of the above (14) Other (please specify)_________
Who is primarily parenting the child in your story? (pick up to three)	(1) Mother (2) Father (3) Step mother (4) Step father (5) Sister(s) (6) Brother(s) (7) Maternal grandparents (8) Paternal grandparents (9) Other family member (10) Other non-family member (11) Child is an orphan (12) Foster parent (13) Other (14) None
With whom of the following does the child in your story live? (pick all that apply)	(1) Mother (2) Father (3) Step mother (4) Step father (5) Sister(s) (6) Brother(s) (7) Maternal grandparents (8) Paternal grandparents (9) Other family member (10) Other non-family member (11) Foster parent(s) (12) Other (13) None
Multiple Choice Questions About the Participant	
What is your age (years)?	(1) 12–17 (2) 18–24 (3) 25–34 (4) 35–44 (5) 45–54 (6) 55–64 (7) 65 or older (8) Prefer not to say
Do you self-identify as:	(1) Ethnic minority (2) Indigenous (3) Refugee (4) Internally displaced (5) Economic migrant (6) Conflict-affected (7) Disaster-affected (8) Poverty (9) Person with a disability (10) Person with a physical or mental health condition (11) Unsure (12) None (13) Other (please specify)________
Research Assistant Questions	
Was violence mentioned in the story?	(1) Sexual (2) Physical (3) Domestic (4) Emotional (5) None
Does the story mention any of the following adverse childhood experiences (ACEs)?	(1) Physical neglect (2) Emotional neglect (3) Incarceration (4) Mental health or attempted suicide of parent Substance abuse (5) Loss of parent through divorce/separation/death
Which disability type, if any, was mentioned in the story?	(1) Intellectual (2) Physical (3) Hearing (4) Visual (5) Psychosocial (6) Other (7) None
Is this a story about parenting in adversity?	(1) Yes (2) No (3) Unsure
Where did this interview take place?	(1) Sake (2) Lac Vert (3) Mugunga (4) Nyiragongo
What is the gender of the narrator?	(1) Female (2) Male (3) Other (4) I don’t know
What story number is this for the participant?	1st 2nd 3rd 4th
Comments	
